# The prevalence of traumatic experiences and PTSD according to DSM-5 and ICD-11 in the German general population

**DOI:** 10.1017/S2045796025100164

**Published:** 2025-08-27

**Authors:** Amelie Pettrich, Yuriy Nesterko, Heide Glaesmer

**Affiliations:** 1 Department of Medical Psychology and Medical Sociology, University of Leipzig, Leipzig, Germany; 2 Department for Traumatic Stress and Transcultural Studies, Center ÜBERLEBEN, Berlin, Germany; 3 Department for Clinical Psychological Intervention, Freie University Berlin, Berlin, Germany

**Keywords:** diagnostic criteria, DSM-5, ICD-11, prevalence, PTSD, trauma exposure

## Abstract

**Aims:**

The Diagnostic and Statistical Manual of Mental Disorders – 5th Edition (DSM-5) and International Classification of Diseases – 11th Revision (ICD-11) employ different post-traumatic stress disorder (PTSD) criteria, necessitating updated prevalence estimates. Most of the existing evidence is still based on ICD-Tenth Revision and DSM-Fourth Edition criteria, leading to varied estimates across populations. This study provides current PTSD prevalence rates in the German general population, comparing DSM-5 and ICD-11 criteria and examines variations by age and gender.

**Methods:**

In a 2016 cross-sectional survey of 2404 adults (18–94 years) representative of the German general population, participants completed the Life-Events-Checklist for DSM-5 (LEC-5) for trauma exposure and the PTSD Checklist for DSM-5 (PCL-5) for PTSD symptoms. Probable PTSD diagnoses were based on DSM-5-, ICD-11-algorithms and suggested cut-off scores. Chi-square and McNemar’s tests were used to test differences in prevalence rates by diagnostic framework, age and gender.

**Results:**

Of the total sample, 47.2% (*n* = 1135) reported experiencing at least one lifetime traumatic event (TE), with transportation accidents (7.3%) and life-threatening injuries (4.9%) being most common. Probable PTSD prevalence was 4.7% under both DSM-5 and ICD-11 criteria, and 2.6% based on a conservative cut-off normed for prevalence estimation. Gender and age were not significantly associated with TE exposure or PTSD prevalence, though trauma types varied: female participants more often reported sexual violence and severe suffering, while more male participants reported physical assaults and various types of accidents. DSM-5 and ICD-11 diagnostic algorithms had substantial yet not perfect agreement (*κ* = 0.62). Particularly within the re-experiencing symptoms, cluster agreement was only moderate (*κ* = 0.57). The cut-off method aligned more closely with DSM-5 (*κ* = 0.60) than ICD-11 algorithm (*κ* = 0.42).

**Conclusions:**

This study provides updated PTSD prevalence estimates for the German general population and underscores differences between DSM-5 and ICD-11 in identifying cases, particularly with respect to re-experiencing symptoms. These findings emphasize that while overall PTSD prevalence rates under DSM-5 and ICD-11 criteria are similar, the diagnostic frameworks identify partially distinct cases, reflecting differences in symptom definitions. This highlights the need to carefully consider the impact of evolving diagnostic criteria when interpreting prevalence estimates and comparing results across studies.

## Introduction

Recent updates in diagnostic criteria in Diagnostic and Statistical Manual of Mental Disorders – 5th Edition (DSM-5; American Psychiatric Association, 2013) and International Classification of Diseases – 11th Revision (ICD-11; World Health Organization, 2022) have refined the understanding and identification of post-traumatic stress disorder (PTSD). These revisions reflect evolving perspectives on the disorder and have introduced notable divergences between the two systems. Consequently, there is a pressing need to generate new prevalence data, as most existing studies still rely on ICD-Tenth Revision (ICD-10) and DSM-Fourth Revision (DSM-IV) criteria. Valid epidemiological data are necessary to quantify the impact of PTSD and are critical for estimating treatment needs and informing public health planning. We aim to present updated prevalence data on PTSD in the German general population, which will help clarify the divergences in diagnostic operationalization between ICD-11 and DSM-5 and their impact on case identification.

Data of the WHO World Mental Health Surveys indicate an average lifetime prevalence of PTSD of 3.9% in the general population worldwide and 5.6% among those exposed to trauma (Koenen *et al.*, 2017). However, Atwoli *et al.* (2015) report that global lifetime prevalence rates for PTSD vary widely, ranging from 1.3% to 12.2%. In European Countries, prevalence rates range from 1% to 3%, with distinct variation between countries (Trautmann and Wittchen, 2018). Given the variability in prevalence rates across countries, national studies are crucial for obtaining precise data to inform national healthcare policies. Studies regarding the prevalence of PTSD in Germany show varying results between 0.7% and 3.9% depending on the population under study, diagnostic methods and the time frame considered. Regarding 1-month prevalence, several studies using DSM-IV criteria have produced estimates between 1.5% and 3.9%. For instance, Spitzer *et al.* (2008) reported a prevalence of 1.5% in a predominantly elderly population, sampled between 2002 and 2006, using SCID interviews and the CIDI screener. Maercker *et al.* (2008) observed 2.3% in a general population sample (2005) using modified Perceived Stress Scale questionnaires. Glaesmer *et al.* (2010) reported 3.9% in a population aged 60 and older (sampled in 2008) based on self-report (Posttraumatic Stress Diagnostic Scale, PDS). Lukaschek *et al.* (2013) reported 1.7% in a regional sample from southern Germany (2006–2008), combining interviews and self-report (Impact of Event Scale, PDS), while Hauffa *et al.* (2011) found 2.9% in the general population, sampled in 2007 with only self-report (PDS). More recently, Maercker *et al.* (2018) reported a 1-month prevalence of 1.5% using ICD-11 criteria and the International Trauma Questionnaire (ITQ) in a general population sample from 2016. Regarding 12-month and lifetime prevalence of PTSD in the German general population, earlier studies based on DSM-IV criteria and various assessment methods reported 12-month prevalence rates ranging from 0.7% to 2.3% (Jacobi *et al.*, 2014; Perkonigg *et al.*, 2000; Schmidt *et al.*, 2013) and lifetime prevalence rates between 1.2% and 3.1% (Perkonigg *et al.*, 2000; Spitzer *et al.*, 2008). PTSD prevalence estimates using DSM-5 criteria for the German general population are lacking so far. The studies mentioned above highlight the variability in PTSD prevalence estimates, influenced by the population under study, the diagnostic framework (DSM-IV vs. ICD-10/-11) and the data collection methods (interview vs. self-report). To ensure accurate estimates, it is crucial to address each of these aspects.

One aspect influencing prevalence estimates is the specific population and geographic and political context under study, as trauma exposure rates and sample characteristics vary significantly across settings. The frequency of potentially traumatizing events like natural disasters or armed conflicts differs across regions and historical periods (Atwoli *et al.*, 2015; Koenen *et al.*, 2017). Additionally, different prevalence rates are observed based on the composition of the sample, whether clinical, trauma-exposed, military or general population (Trautmann and Wittchen, 2018). Cohort effects within Germany, attributable to the World War II generation, further contribute to variability (Maercker *et al.*, 2008). Consequently, findings are diverse and often confined to the specific sample under examination, precluding broad generalizations.

Another important factor affecting PTSD prevalence estimates is the diagnostic framework used, as DSM-5 and ICD-11 both provide standardized criteria to facilitate diagnosis, yet significant differences exist between the two. The DSM-5 categorizes 20 symptoms into four clusters (re-experiencing, avoidance of reminders of trauma, negative alterations in cognition and mood, heightened arousal or reactivity) (American Psychiatric Association, 2013). The ICD-11 clusters only six symptoms into three categories (re-experiencing/avoidance of reminders of the trauma/sense of threat) (World Health Organization, 2022). Both frameworks require symptoms to persist and to significantly impair social, occupational or other important areas of functioning, yet the DSM-5 offers a more structured outline of symptom duration (DSM-5: more than a month, ICD-11: several weeks). The differences in operationalization lead to discrepancies in PTSD case identification between the two diagnostic frameworks, underscoring the need for further exploration into factors influencing diagnostic decisions. In addition, the definitions of what constitutes a traumatic event (TE) differ slightly: DSM-5 emphasizes objective threat and exposure routes, whereas ICD-11 focuses more on the subjective experience of extreme threat. Research using various assessment tools across different populations consistently shows lower PTSD prevalence rates according to ICD-11 compared to DSM-5 (Hansen *et al.*, 2015; Heeke *et al.*, 2020; Hyland *et al.*, 2016; O’Donnell *et al.*, 2014; Wisco *et al.*, 2016), with only one study reporting slightly higher prevalence rates of PTSD according to ICD-11 (Stein *et al.*, 2014). Studies find that more individuals meet the re-experiencing and hyperarousal criteria for DSM-5 compared to ICD-11 (Hyland *et al.*, 2016; O’Donnell *et al.*, 2014; Schellong *et al.*, 2019; Wisco *et al.*, 2016). Specifically, intrusive memories play a crucial role in this disparity, with studies showing that additionally including intrusive memories in ICD-11 criteria aligns prevalence rates more closely with DSM-5 (O’Donnell *et al.*, 2014; Schellong *et al.*, 2019).

In addition to population and diagnostic framework, the method of data collection (the use of clinical interviews versus self-report instruments) can can substantially influence prevalence estimation. Clinical interviews are considered the gold standard for their accuracy but are challenging to implement on large-scale samples. Self-report instruments, though more feasible for widespread use and screening, only provide probable PTSD diagnosis (Bovin and Marx, 2023). Beyond the use of diagnostic algorithms based on self-report instruments, empirically derived cut-off-scores are a common procedure for case identification. However, when used for prevalence estimation, they require adjustments and critical reflection to ensure accuracy (Terhakopian *et al.*, 2008). Cut-off scores, optimized for sensitivity, are lenient regarding false positives, potentially leading to overestimation of positive cases, especially in populations with a low PTSD base rate. For example, in a population of 1000 people with only 4 true PTSD cases (0.4% prevalence), a test with perfect sensitivity (1.0) and 75% specificity would incorrectly identify 249 individuals as positive (25% of the 996 true negatives). This results in an inflated prevalence estimate of 25.3%. Therefore, prevalence estimates using self-report measures in populations with low base rates of PTSD, such as the general population, require careful evaluation of existing cut-off values to prevent overestimation. When self-report instruments are used without a clinical interview, it is recommended to apply a cut-off value established through comparison with clinical interview outcomes in an independent sample, prioritizing a high positive predictive value and accurate estimation of true prevalence (Pettrich *et al.*, 2025; Terhakopian *et al.*, 2008).

Beyond these factors, stratification by age and gender appears to be crucial in understanding PTSD risk. Previous research suggests that women may be at a higher risk of developing PTSD, particularly in response to trauma types like sexual violence (Kessler *et al.*, 2014; Tolin and Foa, 2008). However, this gender difference seems to be influenced by various mediating factors, such as pre-existing mental health conditions and neurobiological responses (Kornfield *et al.*, 2018; McLean and Anderson, 2009). Age-related risk factors also seem to play a role, with younger individuals possibly facing higher risk due to trauma experienced during vulnerable developmental stages (Teicher *et al.*, 2018). Conversely, for older adults, PTSD may arise from the cumulative impact of lifetime trauma, such as war-related experiences, although PTSD is often underreported in this population due to age-related vulnerabilities (Brewin *et al.*, 2000; Frans *et al.*, 2005; Ogle *et al.*, 2014). These gender- and age-related disparities in PTSD risk underscore the multifactorial nature of trauma response, a central theme in the present study’s investigation of PTSD prevalence across different demographic groups.

The present study examines the prevalence of TEs and probable PTSD in a representative sample of the general German population and aims to assess the comparability of PTSD prevalence rates based on DSM-5 and ICD-11 criteria. We use a widely recognized self-report screening tool and apply various diagnostic algorithms to evaluate PTSD criteria. While ICD-11 distinguishes between PTSD and Complex PTSD (CPTSD), our study focused exclusively on core PTSD symptoms as operationalized by DSM-5 and ICD-11 definitions. Additionally, the study investigates trauma and PTSD prevalence stratified by age and gender, exploring disparities and risk factors within the German general population.

## Methods

### Subjects

This cross-sectional study is part of a representative survey of the general German population conducted in 2016 by an independent market and social research institute (USUMA, Berlin). The sampling procedure is described elsewhere in more detail (Pettrich *et al.*, 2024). In total, 2510 people (aged 14–94 years) filled out the self-report questionnaire given to them. After excluding 86 underage participants and 20 subjects with missing data on all items of the PTSD Checklist for DSM-5 (PCL-5) and Life-Events-Checklist for DSM-5, the final sample consisted of 2404 participants aged 18–94 years. Characteristics of the study sample are presented in Table 1. In our pursuit to derive prevalence estimates based on the PCL-5, we used the weighted sample. The resulting analysis sample was representative regarding age (*M* = 50.24; *SD* = 18.07) and gender for the Federal Republic of Germany when compared to the microcensus of the Federal Office of Statistics of the sampling year. The Ethics Committee of the Medical Faculty of the University of Leipzig determined the proposed study to be outside their scope but expressed no ethical concerns for the survey.

**Table 1.** Socio-demographic characteristics in the sample (*N* = 2404)

		*n*	%
Gender	Male	1174	48.8
	Female	1230	51.2
Age, years	18−30	463	19.3
	31−50	700	29.1
	51−70	840	34.9
	>71−94	401	16.7
Education	No higher education entrance qualification	1760	73.2
	A-levels/higher education entrance qualification	395	16.4
	University degree	243	10.1
	Missing data	6	0.2
Household income (per month), €	<1250	286	11.9
	1250–2500	905	37.6
	>2500	1111	46.2
	Missing data	102	4.2
Residence	Rural area	300	12.5
	Urban area	2104	87.5
Partner	Living with partner	1540	64.1
	Living without partner	846	35.2
	Missing data	18	0.7

### Instruments

**The LEC-5** (Krüger-Gottschalk *et al.*, 2017; Weathers *et al.*, 2013) is a self-report measure assessing the lifetime prevalence of TEs. Sixteen predefined TEs and the option to describe any other very stressful event or experience not listed before are presented. The A-criterion of the PTSD diagnosis is endorsed when at least one of the events is answered by ‘Happened to me’, ‘Witnessed it’ and ‘Part of my job’. The answer options ‘Heard about it’, ‘Not sure’ and ‘Doesn’t apply’ are not considered affirmative. Although originally developed for DSM-5, the LEC-5 is widely used in ICD-11 PTSD research to ensure a consistent assessment of the A-criterion (e.g., Cloitre *et al.*, 2018).

**The PCL-5** (Krüger-Gottschalk *et al.*, 2017; Weathers *et al.*, 2013) consists of 20 items covering the DSM-5 PTSD symptoms. Symptom severity in the past month can be rated on a 5-point Likert scale from 0 (‘Not at all’), 1 (‘A little bit’), 2 (‘Moderately’), 3 (‘Quite a bit’) to 4 (‘Extremely’). Following the DSM-5 diagnostic criteria, a diagnosis can be derived if one re-experiencing symptom, one avoidance symptom, two negative alterations in thinking and mood symptoms and two heightened arousal or reactivity symptoms are endorsed (American Psychiatric Association, 2013). Although the PCL-5 has been originally developed to assess DSM-5 criteria, a subset of items was created in accordance with previous research (Heeke *et al.*, 2022, 2020; Kuester *et al.*, 2017; Schellong *et al.*, 2019) to assess ICD-11 diagnostic criteria (items 2, 3, 6, 7, 17, 18) and a probable PTSD diagnosis can be given if one re-experiencing, one avoidance of reminders of the trauma and one sense of threat symptom are fulfilled (World Health Organization, 2022). Based on an empirically derived cut-off score, the PCL-5 screens positive for PTSD if the sum score of all items exceeds a cut-off score of 31–33 in the German population for clinical use (Krüger-Gottschalk *et al.*, 2017) and of 38 for prevalence estimation (sensitivity: 0.84, specificity: 0.703) (Pettrich *et al.*, 2025).

### Statistical procedure and considerations

All analyses were conducted using R (R Core Team, 2021). In this data set, in 4.83% of the subjects, the answers were incomplete (with a total of 0.6% missing data points throughout the whole data set). Little’s missing completely at random test indicated that data were missing systematically (*χ*^2^(874) = 1263.7, *p* < .001). Therefore, the missing data treatment used the predictive mean matching method of the mice package (Buuren and Groothuis-Oudshoorn, 2011). We defined the A-criterion for estimating PTSD prevalence as met if participants endorsed at least one of the 16 predefined TEs on the LEC-5. Other stressful events were not included in the A-criterion, since the answers were very heterogenous and did not meet the A-criterion in many cases. Prevalence estimations included frequencies of probable diagnoses based on diagnostic algorithms according to DSM-5 and ICD-11 and a cut-off score of 38 (Pettrich *et al.*, 2025), stratified by age and gender. Trauma frequencies were reported based on the worst event reported, with unspecified TEs or no indicated worst event categorized as ‘Other Stressful Event/Not Specified’. Chi-square tests with simulated *p*-values (Monte Carlo method) were used for difference testing of stratified prevalences where expected cell counts were low. McNemar’s test for paired samples was employed to compare case identification across different diagnostic frameworks.

## Results

A total of 1135 subjects (47.21%) of the sample reported at least one TE. Of these, 438 (18.22%) had only one TE, 569 (23.67%) had between two and five, and 128 (5.32%) reported more than five. The most common TEs were transportation accident (*n* = 175, 7.28%), life-threatening illness/injury (*n* = 118, 4.91%), severe suffering (*n* = 109, 4.53%) and serious accident (*n* = 76, 3.16%). A total of 573 (48.81%) male and 562 (45.69 %) female subjects reported at least one TE. Overall exposure to at least one TE did not differ between gender (*χ*^2^ = 2.34, *p* = 0.13) or age groups (*χ*^2^ = 7.06, *p* = 0.072). Certain TEs were reported significantly more often by female subjects, including natural disasters, sexual assaults, unwanted sexual experiences and severe suffering. In contrast, male subjects reported higher exposure to fire/explosion, transportation accidents, serious accidents and physical assault. Older adults were more likely to report war-related TEs (for detailed information and test statistics, see Table 2).

**Table 2.** Prevalence and characteristics of index TE stratified by gender and age

TE	TE descriptives				Trauma prevalence stratified by gender					Trauma prevalence stratified by age								
					Male		Female			18−30 yrs		31−50 yrs		51 yrs		71+ yrs		
	Prevalence		Frequency of the event	Years since exposure	(*n* = 1174)		(*n* = 1230)			(*n* = 463)		(*n* = 700)		(*n* = 840)		(*n* = 401)		
	*n*	%	M (SD)	M (SD)	*n*	%	*n*	%	*χ*^2^	*n*	%	*n*	%	*n*	%	*n*	%	*χ*^2^
Any TE^a^	1135	47.21	1.47 (1.63)	16.63 (16.53)	573	48.81	562	45.69	2.34	217	46.87	304	43.43	410	48.81	204	50.87	7.06
Natural disasters	51	2.12	1.02 (0.16)	15.92 (16.21)	18	1.53	33	2.68	4.41*	6	1.3	10	1.43	20	2.38	15	3.74	8.69*
Fire/Explosion	44	1.83	1.02 (0.16)	17.05 (15.63)	35	2.98	9	0.73	15.36***	13	2.81	17	2.43	10	1.19	4	1	8.18*
Transportation accident	175	7.28	1.15 (0.66)	16.62 (14.72)	113	9.63	62	5.04	14.86***	37	7.99	42	6	73	8.69	23	5.74	30.51***
Serious accident	76	3.16	1 (0)	18.27 (15.11)	59	5.03	17	1.38	23.21***	11	2.38	22	3.14	22	2.62	21	5.24	4.53
Toxic substance exposure	4	0.17	14 (1.73)	19.33 (24.83)	4	0.34	–	–	4	2	0.43	–	–	–	–	2	0.5	4
Physical assault	42	1.75	1.67 (2.83)	13.85 (10.33)	29	2.47	13	1.06	6.1*	18	3.89	13	1.86	11	1.31	–	–	16.48***
Assault with weapon	21	0.87	1.42 (0.84)	22.48 (21.57)	15	1.28	6	0.49	**3.86**	7	1.51	11	1.57	–	–	3	0.75	13.1**
Sexual assault	37	1.54	2.59 (3.63)	28.16 (18.19)	3	0.26	34	2.76	25.97***	6	1.3	15	2.14	13	1.55	3	0.75	10.46*
Unwanted sexual experience	7	0.29	1 (0)	23.17 (21.45)	–	–	7	0.57	7*	2	0.43	3	0.43	2	0.24	–	–	2.71
Combat/War-zone exposure	32	1.33	2.75 (2.89)	55.46 (22.57)	15	1.28	17	1.38	0.12	–	–	3	0.43	7	0.83	22	5.49	35.75***
Captivity	1	0.04	1 (-)	71 (-)	1	0.09	–	–	1	–	–	–	–	–	–	1	0.25	3
Life-threatening illness/Injury	118	4.91	1.36 (1.05)	9.96 (10.69)	56	4.77	62	5.04	0.31	20	4.32	23	3.29	54	6.43	21	5.24	27.29***
Severe suffering	109	4.53	1.49 (1.51)	12.77 (12.97)	26	2.21	83	6.75	29.81***	22	4.75	28	4	37	4.4	22	5.49	5.53
Sudden violent death	56	2.33	1.53 (1.58)	14.85 (9.91)	27	2.3	29	2.36	0.07	6	1.3	14	2	26	3.1	10	2.49	16***
Sudden accidental death	59	2.45	1.6 (1.59)	15.29 (12.84)	34	2.9	25	2.03	1.37	2	0.43	23	3.29	22	2.62	12	2.99	19.71***
Caused harm to others	7	0.29	1 (0)	14.71 (9.84)	4	0.34	3	0.24	0.14	–	–	2	0.29	3	0.36	2	0.5	2.71
Other stressful event/Not Specified	790	32.86	1.57 (2.05)	12.67 (13.85)	396	33.73	379	30.81	0.37	162	34.99	234	33.43	259	30.83	120	29.93	63.61***

Abbreviations: *n* = sample size, % = percentage of participants, *χ*^2^ = McNemar’s chi-squared test statistic.

a Any TE: endorsement of at least 1 of the 16 predefined LEC-5 events, excluding ‘other event’ type. Significance Levels: *p < .05 = statistically significant, **p < .01 = highly statistically significant, ***p < .001 = extremely statistically significant.

The prevalence of PTSD in the sample was found to be 4.7% (*n* = 113) according to both the ICD-11 and the DSM-5 diagnostic criteria, 4.08% (*n* = 98) according to the traditional clinical cut-off scoring method (cut-off score = > 33) and 2.62% (*n* = 63) according to the prevalence cut-off scoring method (cut-off score = > 38), we favoured in this study. No significant differences were found for gender (DSM-5: *χ*^2^(1) = 0.004, *p* = .951; ICD-11: *χ*^2^(1) = 1.843, *p* = .175; cut-off: *χ*^2^(1) = 0.696, *p* = 0.404) or age groups (DSM-5: *χ*^2^(3) = 3.598, *p* = .308; ICD-11: *χ*^2^(3) = 1.265, *p* = .737; cut-off: *χ*^2^(3) = 4.01, *p* = .261) for any of the prevalence estimates. Probable PTSD prevalence in trauma-exposed individuals (*n* = 1135) ranged between 5.55% (cut-off scoring method) and 9.96% (DSM-5 and ICD-11 scoring method). Higher conditional PTSD prevalence rates were observed in subgroups exposed to sexual (16.22–32.43%) and physical assaults (2.38–21.43%), unwanted sexual experiences (14.29%) and causing harm to others (14.29%) depending on the diagnostic framework (see Table 3).

**Table 3.** PTSD prevalence by index TE and comparison of DSM-5, ICD-11 and cut-off case identification

Trauma	TE prevalence		Event-specific conditional PTSD prevalence						Agreement in case identification^b^					
			ICD − 11		DSM − 5		Cut-off (38)		DSM − 5/ICD − 11		DSM − 5/Cut-off (38)		ICD − 11/Cut-off (38)	
	*n*	%	*n*	%	*n*	%	*n*	%	%	*χ*^2^	%	*χ*^2^	%	*χ*^2^
Any TE^a^	1135	47.21	113	9.96	113	9.96	63	5.55	48.68	0	45.45	36.38***	30.37	25.54***
Natural disasters	51	2.12	2	3.92	4	7.84	0	0	50	0.5	0	2.25	0	0.5
Fire/Explosion	44	1.83	2	4.55	2	4.55	1	2.27	33.33	0	50	0	0	0
Transportation accident	175	7.28	8	4.57	12	6.86	5	2.86	66.67	2.25	30.77	4*	30	0.57
Serious accident	76	3.16	5	6.58	7	9.21	4	5.26	33.33	0.17	57.14	1.33	0	0
Toxic substance exposure	4	0.17	–	–	–	–	0	0	–	–	–	–	–	–
Physical assault	42	1.75	7	16.67	9	21.43	1	2.38	77.78	0.5	11.11	6.12*	14.29	4.17*
Assault with weapon	21	0.87	–	–	–	–	0	0	–	–	–	–	–	–
Sexual assault	37	1.54	12	32.43	8	21.62	6	16.22	53.85	1.5	75	0.5	50	4.17*
Unwanted sexual experience	7	0.29	1	14.29	1	14.29	1	14.29	100	–	100	–	100	–
Combat/War-zone exposure	32	1.33	3	9.38	3	9.38	3	9.38	100	–	100	–	100	–
Captivity	1	0.04	–	–	–	–	0	0	–	–	–	–	–	–
Life-threatening illness/Injury	118	4.91	13	11.02	11	9.32	8	6.78	9.09	0.05	35.71	0.44	5	0.84
Severe suffering	109	4.53	15	13.76	14	12.84	8	7.34	61.11	0	57.14	4.17*	35.29	3.27
Sudden violent death	56	2.33	4	7.14	2	3.57	2	3.57	20	0.25	100	–	20	0.25
Sudden accidental death	59	2.45	4	6.78	4	6.78	1	1.69	33.33	0	0	0.8	0	0.8
Caused harm to others	7	0.29	1	14.29	1	14.29	1	14.29	100	–	100	–	100	–
Other stressful event/Not specified	790	32.86	36	4.56	56	7.09	22	2.78	41.03	0.54	31.15	29.17***	32.14	22.13***

Abbreviations: *n* = sample size, % = percentage of participants, M (SD) = Mean (standard deviation), *χ*^2^ = McNemar’s chi-squared test statistic.

a Any TE: endorsement of at least 1 of the 16 predefined LEC-5 events, excluding ‘other event’ type.

b Agreement in case identification: the percentage reflects the agreement of positive cases identified by the diagnostic systems, relative to those identified as positive by either system, indicating diagnostic concordance. McNemar χ^2^ tests assess whether differences in identification between the diagnostic systems are statistically significant.

* Significance Levels: *p* < .05 = statistically significant, **p < .01 = highly statistically significant, ***p < .001 = extremely statistically significant.

While overall PTSD prevalence rates were identical between the DSM-5 and ICD-11 criteria, the two frameworks identified partially different cases (see Table 3). The agreement between the DSM-5 and ICD-11 criteria was substantial, though not perfect (Cohen’s *κ* = 0.62; 95% CI [0.54, 0.69]). Of the subjects who screened positive for probable PTSD according to either diagnostic algorithm, 48.68% were identified by both systems. McNemar’s test showed no significant difference between the two diagnostic criteria (*χ*^2^(1) = 0, *p* = 1.00), suggesting that the discrepancies in case identification were evenly distributed between the two systems.

Upon closer examination of the symptom clusters, significant differences were found in the re-experiencing criteria between ICD-11 and DSM-5. Cohen’s kappa for the re-experiencing criteria was moderate (*κ* = 0.57; 95% CI [0.52, 0.61]). Of the subjects who screened positive in the re-experiencing symptom cluster according to either system, 52.18% were identified by both diagnostic frameworks. McNemar’s chi-square test showed a significant difference in the proportions of cases identified (*χ*^2^(1) = 217, *p* < 0.001). In contrast, no significant differences were found in the fulfillment of the combined D- and E-criteria of the DSM-5 and the Sense of Threat criteria of the ICD-11.

The cut-off scoring method (cut-off score>38) showed higher agreement with the DSM-5-algorithm (Cohen’s *κ* = 0.60, 95% CI [0.51, 0.68]) than with the ICD-11-algorithm (Cohen’s *κ* = 0.42, 95% CI [0.33, 0.52]). Of the subjects who screened positive for PTSD by either the diagnostic algorithm or the cut-off scoring method, 45.45% were identified by both the DSM-5 and the cut-off method, while 30.37% were identified by both the ICD-11 and the cut-off method. For more detailed analyses of symptom clusters and contingency tables, see appendix.

## Discussion

In large-scale population studies, it is often not feasible to conduct clinical interviews. Moreover, it is well-known that in samples with relatively low base rates of PTSD, self-report measures tend to overestimate prevalence rates. Therefore, in this study, we estimated the prevalence of PTSD in the German general population using a cut-off value specifically designed for prevalence estimation, as well as commonly used diagnostic algorithms based on DSM-5 and ICD-11 criteria. In addition to providing an updated prevalence estimate, we also aimed to further investigate the comparability of the DSM-5 and ICD-11 frameworks. Our findings yield several important conclusions.

Firstly, approximately 2.62% of the population screened positive for PTSD using a conservative cut-off (cut-off score = 38), whereas prevalence estimates reached up to 4.7% when applying the more lenient DSM-5/ICD-11 diagnostic algorithm. Additionally, almost half of the German general population has experienced at least one TE. Importantly, demographic factors such as age and gender did not significantly affect overall trauma exposure or PTSD diagnostic status, though specific trauma types were unevenly distributed. Women were more likely to report sexual assault and severe suffering, while men reported higher exposure to fire or explosions, transportation accidents, serious accidents and physical assault. In terms of PTSD prevalence comparability, although both DSM-5 and ICD-11 yielded identical prevalence rates, they identified partially different individual cases. With only about half of the identified cases supported by both diagnostic frameworks, it is evident that while both systems capture similar proportions of PTSD cases, their diagnostic criteria prioritize particularly the re-experiencing cluster differently. Furthermore, the comparison of DSM-5 and ICD-11 with the cut-off-scoring method revealed that DSM-5 had a higher agreement with the cut-off-based procedure (cut-off 38) than ICD-11. This highlights the need for continued refinement of diagnostic algorithms to ensure accurate identification of PTSD cases across varying assessment methods. Lastly, our findings indicate that using the PCL-5 as a self-report instrument, normed against a clinical interview for clinical purposes (diagnostic algorithms, cut-off = 33), results in much higher PTSD prevalence rates compared to using the same instrument with a cut-off value specifically designed for prevalence estimation (cut-off = 38), where the rates align more closely with expected prevalence.

The prevalence rates in our study are slightly higher than those reported in previous research. Even using more conservative, cut-off scores adjusted for prevalence estimation, our rates are on the higher end of those found in similar populations, such as 2.9% by Hauffa *et al.* (2011) and 1.6% by Maercker *et al.* (2018). The reasons for this upward trend remain unclear, but one potential explanation could be the different diagnostic systems used in the previous studies – with Hauffa *et al.* (2011) using DSM-IV criteria and the PDS and Maercker *et al.* (2018) using the ITQ based on ICD-11 criteria. Our findings contrast with much of the existing literature, where most studies report higher 1month PTSD prevalence rates using DSM-5 compared to ICD-11 criteria, while we observed equivalent rates with substantial agreement for both. Several studies, such as Hansen *et al.* (2015) and Hyland *et al.* (2017), found lower PTSD diagnostic rates based on ICD-11 criteria in Danish (via Harvard Trauma Questionnaire symptom mapping) and British (ITQ and PCL-5) populations. Similarly, Heeke *et al.* (2020) reported that the DSM-5 criteria identified more individuals with PTSD among traumatized refugees (PCL-5 symptom mapping). O’Donnell *et al.* (2014) observed DSM-5 producing twice the PTSD prevalence (6.7%) compared to ICD-11 (3.3%) via the Clinician-Administered PTSD Scale interview, and Wisco *et al.* (2016) noted ICD-11 yields 10-30% lower prevalence rates due to its stricter symptom criteria (National Stressful Events Survey symptom mapping). Our results, however, align with studies like Morina *et al.* (2014), who found no significant differences between DSM-IV and ICD-11 in war survivors (PDS symptom mapping), and Kuester *et al.* (2017), where DSM-5 (56%) and ICD-11 (48%) prevalence rates were not statistically different (PCL-5 symptom mapping).

This study has several notable strengths. First, it utilizes a large, representative dataset, making it the first German PTSD prevalence study to assess DSM-5 criteria and directly comparing prevalence rates according to ICD-11 and DSM-5 in the general population. Our findings contrast with previous studies that typically reported higher PTSD prevalence under DSM-5 criteria; this effect was not observed in our sample among individuals with at least one TE. Additionally, the study demonstrates that despite showing substantial agreement, DSM-5 and ICD-11 criteria identify partially different groups of individuals with PTSD. The re-experiencing cluster seems to play a major role here. This finding highlights the importance of understanding how different diagnostic criteria may influence case identification. Finally, this study introduces a novel approach by using a cut-off for prevalence estimation (Pettrich *et al.*, 2025) on a self-report instrument to mitigate the tendency for inflated results commonly associated with self-reports. Despite these strengths, there are limitations to consider. Notably, the ICD-11 measure was adapted from an instrument originally developed for DSM-5 criteria. Consequently, the findings and implications regarding the diagnostic performance of ICD-11 criteria are specific to this operationalization. Additionally, it should be considered when interpreting diagnostic comparisons, that trauma exposure in this study was assessed using the DSM-oriented LEC-5. An important limitation of our study is that it does not differentiate between PTSD and CPTSD as defined in ICD-11. Our ICD-11 approximation was based on selected PCL-5 items and did not assess Disturbances in Self-Organization, a key criterion for CPTSD. Therefore, some individuals who would meet criteria for CPTSD may be included in our ICD-11 PTSD estimate. Similarly, the dissociative subtype of PTSD described in DSM-5 was not assessed, as the PCL-5 does not include items related to depersonalization or derealization. While trauma types commonly associated with CPTSD were reported by a minority of the sample, we cannot exclude the possibility that a proportion of CPTSD cases are misclassified. Future prevalence studies should incorporate validated instruments such as the ITQ to capture both PTSD and CPTSD as defined in ICD-11. Additionally, both diagnostic systems were applied only at the symptom cluster level, and we did not account for the time criterion, subjective impairment or exclusion criteria of substance use or physical factors due to a lack of available information. The absence of a clinical interview restricts our conclusions to PTSD screening diagnoses. Furthermore, the suggested cut-off of 38 for prevalence estimation was normed on a clinical sample with a higher PTSD base rate, which may limit its applicability to the general population. Additionally, a potential limitation of our study could be that we did not exclude participants with cognitive impairments, which may have affected the accuracy of their self-report responses.

Future research should explore the mechanisms behind the identification of different cases at the symptom cluster level. Moreover, prevalence studies using clinical interviews should be conducted in the general German population to further validate our findings. A meta-analysis could also be beneficial to integrate existing knowledge from the growing body of prevalence studies conducted on various samples within the German population and beyond.

## Supporting information

10.1017/S2045796025100164.sm001 Pettrich et al. supplementary material Pettrich et al. supplementary material

## Data Availability

The data are not publicly available due to the informed consent given by the participants.

## Supplementary material

The supplementary material for this article can be found at https://doi.org/10.1017/S2045796025100164.

## References

[ref1] American Psychiatric Association (2013) Diagnostic and Statistical Manual of Mental Disorders. American Psychiatric Association. 10.1176/appi.books.9780890425596 (accessed 17 February 2025).

[ref2] Atwoli L, Stein DJ, Koenen KC and McLaughlin KA (2015) Epidemiology of posttraumatic stress disorder. *Current Opinion in Psychiatry* 28(4), 307–311. doi:10.1097/YCO.0000000000000167 26001922 PMC4452282

[ref3] Bovin MJ and Marx BP (2023) The problem with overreliance on the PCL–5 as a measure of PTSD diagnostic status. *Clinical Psychology: Science and Practice* 30(1), 122–125. doi:10.1037/cps0000119

[ref4] Brewin CR, Andrews B and Valentine JD (2000) Meta-analysis of risk factors for posttraumatic stress disorder in trauma-exposed adults. *Journal of Consulting and Clinical Psychology* 68(5), 748–766. doi:10.1037/0022-006X.68.5.748 11068961

[ref5] Buuren SV and Groothuis-Oudshoorn K (2011) Mice: multivariate imputation by chained equations in R. *Journal of Statistical Software*. doi: 10.18637/jss.v045.i03.

[ref6] Cloitre M, Shevlin M, Brewin CR, Bisson JI, Roberts NP, Maercker A, Karatzias T and Hyland P (2018) The International Trauma Questionnaire: development of a self-report measure of ICD-11 PTSD and complex PTSD. *Acta Psychiatrica Scandinavica* 138(6), 536–546. doi:10.1111/acps.12956 30178492

[ref7] Frans Ö, Rimmö PA, Åberg L and Fredrikson M (2005) Trauma exposure and post‐traumatic stress disorder in the general population. *Acta Psychiatrica Scandinavica* 111(4), 291–290. doi:10.1111/j.1600-0447.2004.00463.x 15740465

[ref8] Glaesmer H, Gunzelmann T, Braehler E, Forstmeier S and Maercker A (2010) Traumatic experiences and post-traumatic stress disorder among elderly Germans: results of a representative population-based survey. *International Psychogeriatrics* 22(4), 661–670. doi:10.1017/S104161021000027X 20353625

[ref9] Hansen M, Hyland P, Armour C, Shevlin M and Elklit A (2015) Less is more? Assessing the validity of the ICD-11 model of PTSD across multiple trauma samples. *European Journal of Psychotraumatology* 6(1). doi: 10.3402/ejpt.v6.28766. PMC4598338 26450830

[ref10] Hauffa R, Rief W, Brähler E, Martin A, Mewes R and Glaesmer H (2011) Lifetime traumatic experiences and posttraumatic stress disorder in the German population: results of a representative population survey. *Journal of Nervous and Mental Disease* 199(12). doi: 10.1097/NMD.0b013e3182392c0d. 22134451

[ref11] Heeke C, Franzen M, Hofmann H, Knaevelsrud C and Lenferink LIM (2022) A latent class analysis on symptoms of prolonged grief, post-traumatic stress, and depression following the loss of a loved one. *Frontiers in Psychiatry* 13, doi:10.3389/fpsyt.2022.878773. PMC9184516 35693969

[ref12] Heeke C, O’Donald A, Stammel N and Böttche M (2020) Same same but different? DSM-5 versus ICD-11 PTSD among traumatized refugees in Germany. *Journal of Psychosomatic Research* 134, 110129. doi:10.1016/j.jpsychores.2020.110129 32413613

[ref13] Hyland P, Shevlin M, Brewin CR, Cloitre M, Downes AJ, Jumbe S, Karatzias T, Bisson JI and Roberts NP (2017) Validation of post‐traumatic stress disorder (PTSD) and complex PTSD using the International Trauma Questionnaire. *Acta Psychiatrica Scandinavica* 136(3), 313–322. doi:10.1111/acps.12771 28696531

[ref14] Hyland P, Shevlin M, McNally S, Murphy J, Hansen M and Elklit A (2016) Exploring differences between the ICD-11 and DSM-5 models of PTSD: does it matter which model is used? *Journal of Anxiety Disorders* 37, 48–53. doi:10.1016/j.janxdis.2015.11.002 26618238

[ref15] Jacobi F, Höfler M, Siegert J, Mack S, Gerschler A, Scholl L, Busch MA, Hapke U, Maske U, Seiffert I, Gaebel W, Maier W, Wagner M, Zielasek J and Wittchen H (2014) Twelve‐month prevalence, comorbidity and correlates of mental disorders in Germany: the Mental Health Module of the German Health Interview and Examination Survey for Adults (DEGS1‐MH). *International Journal of Methods in Psychiatric Research* 23(3), 304–319. doi:10.1002/mpr.1439 24729411 PMC6878234

[ref16] Kessler RC, Rose S, Koenen KC, Karam EG, Stang PE, Stein DJ, Heeringa SG, Hill ED, Liberzon I, McLaughlin KA, McLean SA, Pennell BE, Petukhova M, Rosellini AJ, Ruscio AM, Shahly V, Shalev AY, Silove D, Zaslavsky AM, Angermeyer MC, Bromet EJ, de Almeida JMC, de Girolamo G, de Jonge P, Demyttenaere K, Florescu SE, Gureje O, Haro JM, Hinkov H, Kawakami N, Kovess-Masfety V, Lee S, Medina-Mora ME, Murphy SD, Navarro-Mateu F, Piazza M, Posada-Villa J, Scott K, Torres Y and Carmen Viana M (2014) How well can post-traumatic stress disorder be predicted from pre-trauma risk factors?. *An Exploratory Study in the WHO World Mental Health Surveys’, World Psychiatry* 13(3), 265–274. doi:10.1002/wps.20150 25273300 PMC4219068

[ref17] Koenen KC, Ratanatharathorn A, Ng L, McLaughlin KA, Bromet EJ, Stein DJ, Karam EG, Meron Ruscio A, Benjet C, Scott K, Atwoli L, Petukhova M, Lim CCW, Aguilar-Gaxiola S, Al-Hamzawi A, Alonso J, Bunting B, Ciutan M, de Girolamo G, Degenhardt L, Gureje O, Haro JM, Huang Y, Kawakami N, Lee S, Navarro-Mateu F, Pennell B-E, Piazza M, Sampson N, ten Have M, Torres Y, Viana MC, Williams D, Xavier M and Kessler RC (2017) Posttraumatic stress disorder in the world mental health surveys. *Psychological Medicine* 47(13), 2260–2274. doi:10.1017/S0033291717000708 28385165 PMC6034513

[ref18] Kornfield SL, Hantsoo L and Epperson CN (2018) What does sex have to do with it? The role of sex as a biological variable in the development of posttraumatic stress disorder. *Current Psychiatry Reports* 20(6), 39. doi:10.1007/s11920-018-0907-x PMC6354938 29777319

[ref19] Krüger-Gottschalk A, Knaevelsrud C, Rau H, Dyer A, Schäfer I, Schellong J and Ehring T (2017) The German version of the posttraumatic stress disorder checklist for DSM-5 (PCL-5): psychometric properties and diagnostic utility. *BMC Psychiatry.* 17(1), 379. doi:10.1186/s12888-017-1541-6 PMC5704375 29183285

[ref20] Kuester A, Köhler K, Ehring T, Knaevelsrud C, Kober L, Krüger-Gottschalk A, Schäfer I, Schellong J, Wesemann U and Rau H (2017) Comparison of DSM-5 and proposed ICD-11 criteria for PTSD with DSM-IV and ICD-10: changes in PTSD prevalence in military personnel. *European Journal of Psychotraumatology* 8(1). doi: 10.1080/20008198.2017.1386988. PMC5687795 29163862

[ref21] Lukaschek K, Kruse J, Emeny RT, Lacruz ME, von Eisenhart Rothe A and Ladwig K-H (2013) Lifetime traumatic experiences and their impact on PTSD: a general population study. *Social Psychiatry & Psychiatric Epidemiology* 48(4), 525–532. doi:10.1007/s00127-012-0585-7 23007294

[ref22] Maercker A, Forstmeier S, Wagner B, Glaesmer H and Brähler E (2008) Posttraumatische Belastungsstörungen in Deutschland. *Der Nervenarzt* 79(5), 577–586. doi:10.1007/s00115-008-2467-5 18437339

[ref23] Maercker A, Hecker T, Augsburger M and Kliem S (2018) ICD-11 prevalence rates of posttraumatic stress disorder and complex posttraumatic stress disorder in a German nationwide sample. *Journal of Nervous and Mental Disease* 206(4), 270–276. doi:10.1097/NMD.0000000000000790 29377849

[ref24] McLean CP and Anderson ER (2009) Brave men and timid women? A review of the gender differences in fear and anxiety. *Clinical Psychology Review.* 29(6), 496–505. doi:10.1016/j.cpr.2009.05.003 19541399

[ref25] Morina N, van Emmerik AAP, Andrews B and Brewin CR (2014) Comparison of DSM‐IV and Proposed ICD‐11 Formulations of PTSD among civilian survivors of war and war veterans. *Journal of Traumatic Stress* 27(6), 647–654. doi:10.1002/jts.21969 25418442

[ref26] O’Donnell ML, Alkemade N, Nickerson A, Creamer M, McFarlane AC, Silove D, Bryant RA and Forbes D (2014) Impact of the diagnostic changes to post-traumatic stress disorder for DSM-5 and the proposed changes to ICD-11ʹ. *British Journal of Psychiatry* 205(3), 230–235. doi:10.1192/bjp.bp.113.135285 24809400

[ref27] Ogle CM, Rubin DC and Siegler IC (2014) Cumulative exposure to traumatic events in older adults. *Aging and Mental Health* 18(3), 316–325. doi:10.1080/13607863.2013.832730 24011223 PMC3944195

[ref28] Perkonigg A, Kessler RC, Storz S and Wittchen H (2000) Traumatic events and post‐traumatic stress disorder in the community: prevalence, risk factors and comorbidity. *Acta Psychiatrica Scandinavica* 101(1), 46–59. doi:10.1034/j.1600-0447.2000.101001046.x 10674950

[ref29] Pettrich A, Friedrich M, Nesterko Y and Glaesmer H (2024) The German PCL-5: evaluating structural validity in a large-scale sample of the general German population. *European Journal of Psychotraumatology* 15(1). doi: 10.1080/20008066.2024.2317055. PMC10883083 38379510

[ref30] Pettrich A, Schellong J, Dyer A, Ehring T, Knaevelsrud C, Krüger-Gottschalk A, Nesterko Y, Schäfer I and Glaesmer H (2025) Beyond one-cutoff-fits-all: determining cutoff values for the PTSD checklist for DSM-5 (PCL-5). *European Journal of Psychotraumatology* 16(1). doi: 10.1080/20008066.2025.2514878. PMC12210402 40586325

[ref31] R Core Team (2021) ‘R: a language and environment for statistical computing’. Vienna, Austria: R Foundation for Statistical Computing. https://www.R-project.org/ (accessed 17 February 2025).

[ref32] Schellong J, Hanschmidt F, Ehring T, Knaevelsrud C, Schäfer I, Rau H, Dyer A and Krüger-Gottschalk A (2019) Diagnostik der PTBS im Spannungsfeld von DSM-5 und ICD-11ʹ. *Der Nervenarzt* 90(7), 733–739. doi:10.1007/s00115-018-0668-0 30643956

[ref33] Schmidt C, Watzke A-B, Schulz A, Baumeister S, Freyberger H and Grabe H-J (2013) Die Lebenszeitprävalenz psychischer Störungen in Vorpommern. *Psychiatrische Praxis* 40(04), 192–199. doi:10.1055/s-0033-1343100 23564355

[ref34] Spitzer C, Barnow S, Volzke H, John U, Freyberger HJ and Joergen Grabe H (2008) Trauma and Posttraumatic Stress Disorder in the Elderly. *The Journal of Clinical Psychiatry.* 69(5), 693–700. doi:10.4088/JCP.v69n0501 18452344

[ref35] Stein DJ, McLaughlin KA, Koenen KC, Atwoli L, Friedman MJ, Hill ED, Maercker A, Petukhova M, Shahly V, van Ommeren M, Alonso J, Borges G, de Girolamo G, de Jonge P, Demyttenaere K, Florescu S, Karam EG, Kawakami N, Matschinger H, Okoliyski M, Posada-Villa J, Scott KM, Viana MC and Kessler RC (2014) DSM-5 and ICD-11 definitions of posttraumatic stress disorder: investigating “narrow” and “broad” approaches. *Depression and Anxiety* 31(6), 494–505. doi:10.1002/da.22279 24894802 PMC4211431

[ref36] Teicher MH, Anderson CM, Ohashi K, Khan A, McGreenery CE, Bolger EA, Rohan ML and Vitaliano GD (2018) Differential effects of childhood neglect and abuse during sensitive exposure periods on male and female hippocampus. *NeuroImage* 169, 443–452. doi:10.1016/j.neuroimage.2017.12.055 29288867 PMC5856615

[ref37] Terhakopian A, Sinaii N, Engel CC, Schnurr PP and Hoge CW (2008) Estimating population prevalence of posttraumatic stress disorder: an example using the PTSD checklist. *Journal of Traumatic Stress* 21(3), 290–300. doi:10.1002/jts.20341 18553416

[ref38] Tolin DF and Foa EB (2008) Sex differences in trauma and posttraumatic stress disorder: a quantitative review of 25 years of research. *Psychological Trauma: Theory, Research, Practice, and Policy* 1, 37–85. doi:10.1037/1942-9681.S.1.37 17073529

[ref39] Trautmann S and Wittchen H-U (2018) *Trauma and PTSD in Europe*. (Edited by) Nemeroff C.B., and Marmar C.R. Oxford: Oxford University Press. doi:10.1093/med/9780190259440.003.0008

[ref40] Weathers FW, Litz BT, Keane TM, Palmieri PA, Marx BP and Schnurr PP (2013) *The PTSD Checklist for DSM-5 (PCL-5)*. https://www.ptsd.va.gov/professional/assessment/adult-sr/ptsd-checklist.asp (accessed 30 January 2023).

[ref41] Wisco BE, Miller MW, Wolf EJ, Kilpatrick D, Resnick HS, Badour CL, Marx BP, Keane TM, Rosen RC and Friedman MJ (2016) The impact of proposed changes to ICD-11 on estimates of PTSD prevalence and comorbidity. *Psychiatry Research* 240, 226–233. doi:10.1016/j.psychres.2016.04.043 27124207 PMC4885778

[ref42] World Health Organization (2022) ‘ICD-11: international classification of diseases (11th revision)’. https://icd.who.int/ (accessed 17 February 2025).

