# Supplementary material for: The prevalence of traumatic experiences and PTSD according to DSM-5 and ICD-11 in the German general population
Source: Epidemiol Psychiatr Sci. 2025 Aug 27;34:e44. doi: 10.1017/S2045796025100164 (PMC12450535; doi:10.1017/S2045796025100164)
Supplement: Pettrich et al. supplementary material [file S2045796025100164sup001.docx]

Supplement

# Table of PTSD Prevalence Stratified by Age and Gender Across Diagnostic Methods

|  | total sample | | gender | | | | | Age groups | | | | | | | | |
| --- | --- | --- | --- | --- | --- | --- | --- | --- | --- | --- | --- | --- | --- | --- | --- | --- |
|  | (n=2404) | | male | | female | |  | 18-30 yrs | | 31-50 yrs | | 51-70 yrs | | 70-94 yrs | |  |
|  |  | | (n=1174) | | (n=1230) | |  | (n=463) | | (n=700) | | (n=840) | | (n=401) | |  |
|  | n | % | n | % | n | % | χ2 | n | % | n | % | n | % | n | % | χ2 |
| According to Cutoff (38) | 63 | 2.62 | 27 | 2.3 | 36 | 2.93 | 0.7 | 8 | 1.73 | 15 | 2.14 | 26 | 3.1 | 14 | 3.49 | 4 |
| According to DSM-5 | 113 | 4.7 | 56 | 4.77 | 57 | 4.63 | 0 | 20 | 4.32 | 26 | 3.71 | 48 | 5.71 | 19 | 4.74 | 3.6 |
| Criterion B | 603 | 25.08 | 249 | 21.21 | 354 | 28.78 | 17.92*** | 127 | 27.43 | 130 | 18.57 | 218 | 25.95 | 128 | 31.92 | 27.46*** |
| Criterion C | 467 | 19.43 | 217 | 18.48 | 250 | 20.33 | 1.19 | 91 | 19.65 | 126 | 18 | 164 | 19.52 | 86 | 21.45 | 1.98 |
| Criterion D | 297 | 12.35 | 149 | 12.69 | 148 | 12.03 | 0.18 | 74 | 15.98 | 67 | 9.57 | 104 | 12.38 | 52 | 12.97 | 10.78 |
| Criterion E | 349 | 14.52 | 145 | 12.35 | 204 | 16.59 | 8.34** | 76 | 16.41 | 72 | 10.29 | 124 | 14.76 | 77 | 19.2 | 18.58*** |
| According to ICD-11 | 113 | 4.7 | 50 | 4.26 | 63 | 5.12 | 0.82 | 25 | 5.4 | 31 | 4.43 | 43 | 5.12 | 14 | 3.49 | 2.26 |
| Re-experiencing Criterion | 307 | 12.77 | 134 | 11.41 | 173 | 14.07 | 3.56 | 76 | 16.41 | 72 | 10.29 | 100 | 11.9 | 59 | 14.71 | 11.32 |
| Avoidance Criterion | 467 | 19.43 | 217 | 18.48 | 250 | 20.33 | 1.19 | 91 | 19.65 | 126 | 18 | 164 | 19.52 | 86 | 21.45 | 1.98 |
| Sense of Threat Criterion | 485 | 20.17 | 220 | 18.74 | 265 | 21.54 | 2.76 | 83 | 17.93 | 117 | 16.71 | 189 | 22.5 | 96 | 23.94 | 13.01** |
| Abbreviations: n = sample size, % = percentage of participants, χ² = Chi-squared test statistic. Significance Levels: p < .05 = statistically significant (*), p < .01 = highly statistically significant (**), p < .001 = extremely statistically significant (***). | | | | | | | | | | | | | | | | |

# Appendix Contingency tables

In the following segment, we focus on a subsample of trauma-exposed individuals (n=1135) rather than the total study sample (n=2404).

## Comparison of PTSD Diagnoses Using DSM-5 and ICD-11: Contingency Table Analysis

| DSM-5/ ICD-11 | ICD-11 yes | ICD-11 no |  |
| --- | --- | --- | --- |
| DSM-5 yes | 74 (6.52%) | 39 (3.44%) | 113 (9.96%) |
| DSM-5 no | 39 (3.44%) | 983 (86.61%) | 1022 (90.04%) |
|  | 113 (9.96%) | 1022 (90.04%) | 1135 (100%) |

## Comparison of PTSD Diagnoses Using DSM-5 and Cutoff-Scoring: Contingency Table Analysis

| DSM-5 / Cutoff | Cutoff yes | Cutoff no |  |
| --- | --- | --- | --- |
| DSM-5 yes | 55 (4.85%) | 58 (5.11%) | 113 (9.96%) |
| DSM-5 no | 8 (0.7%) | 1014 (89.34%) | 1022 (90.04%) |
|  | 63 (5.55%) | 1072 (94.45%) | 1135 (100%) |

## Comparison of PTSD Diagnoses Using ICD-11 and Cutoff-Scoring: Contingency Table Analysis

| ICD-11 / Cutoff | ICD-11 yes | ICD-11 no |  |
| --- | --- | --- | --- |
| Cutoff yes | 41 (3.61%) | 22 (1.94%) | 63 (5.55%) |
| Cutoff no | 72 (6.34%) | 1000 (88.11%) | 1072 (94.45%) |
|  | 113 (9.96%) | 1022 (90.04%) | 1135 (100%) |

## Comparison of the re-experiencing symptom cluster between DSM-5 and ICD-11: Contingency Table Analysis

| re-experience criteria | DSM-5 yes | DSM-5 no |  |
| --- | --- | --- | --- |
| ICD-11 yes | 239 (21.06%) | 0 (0%) | 239 (21.06%) |
| ICD-11 no | 219 (19.3%) | 677 (59.65%) | 896 (78.94%) |
|  | 458 (40.35%) | 677 (59.65%) | 1135 (100%) |

Cohen's kappa for the re-experience criteria of the ICD-11 and DSM-5 was 0.57 (95% CI [0.52, 0.61]), indicating moderate agreement. The agreement of the two diagnostic systems was 52.18%. McNemar's chi-squared test resulted in a value of 217 (df = 1, p < 0.001), indicating a significant difference between the paired proportions.

## Comparison of Sense of Threat symptom cluster / combined D&E criteria between DSM-5 and ICD-11: Contingency Table Analysis

| Sense of Threat / combined D&E criteria | DSM-5 yes | DSM-5 no |  |
| --- | --- | --- | --- |
| ICD-11 yes | 249 (21.94%) | 85 (7.49%) | 334 (29.43%) |
| ICD-11 no | 80 (7.05%) | 721 (63.52%) | 801 (70.57%) |
|  | 329 (28.99%) | 806 (71.01%) | 1135 (100%) |

Cohen's kappa was found to be 0.65 (95% CI [0.60, 0.70]), indicating substantial agreement. The agreement of the two measures was 52.18%. Additionally, McNemar's chi-squared test yielded a value of 0.097 (df = 1, p = 0.756), suggesting no significant difference between the paired proportions.
